# Tuberculosis in Canada: 2017

**DOI:** 10.14745/ccdr.v45i23a04

**Published:** 2019-02-07

**Authors:** M LaFreniere, H Hussain, N He, M McGuire

**Affiliations:** 1 Centre for Communicable Disease and Infection Control, Public Health Agency of Canada, Ottawa, ON; 2 Dalla Lana School of Public Health, University of Toronto, Toronto, ON

**Keywords:** tuberculosis, surveillance, incidence rate, TB

## Abstract

**Background:**

Tuberculosis (TB) is a major global health problem that affected an estimated 10 million people worldwide in 2017. The Public Health Agency of Canada monitors active TB disease through a national surveillance system, which is a collaborative effort with the provinces and territories.

**Objective:**

To present an epidemiological summary of active TB cases reported in 2017. Results are discussed in the context of the previous year’s data. Treatment outcomes for cases diagnosed in 2016 are also presented.

**Methods:**

The Canadian Tuberculosis Reporting System is a case-based surveillance system that maintains non-nominal data on people diagnosed with active TB disease in Canada. Data are collected annually from the provinces and territories, analyzed by the Public Health Agency of Canada and validated by each province and territory.

**Results:**

There were 1,796 cases of active TB reported in Canada in 2017 compared with 1,750 cases in 2016, representing a 2.6% increase. There was a corresponding increase in the incidence rate from 4.8 to 4.9 per 100,000 population. Foreign born individuals continued to make up the majority of cases (71.8%) and the incidence rate remained highest among Canadian born Indigenous people (21.5 per 100,000 population), in particular, among the Inuit population (205.8 per 100,000 population). Consistent with the previous decade, TB incidence rates in 2017 continued to be higher among males (5.5 per 100,000) compared with females (4.3 per 100,000), and the majority of cases (45.6%) were between the ages of 15 and 44 years. The incidence rate was highest among adults over 75 years of age (13.8 cases per 100,000 for males and 7.2 for females). Of the TB cases diagnosed in 2016 where outcomes were reported, 80.4% were treated successfully.

**Conclusion:**

Although the incidence rate of TB in Canada in 2017 remained low in the global context and has been relatively stable over the last decade, both the case count and rate have been gradually increasing since 2014. Indigenous and foreign born Canadians continued to be disproportionately represented among TB cases. Canadian TB surveillance data are an important source of information for monitoring progress and informing public health action related to reducing the burden of TB in Canada, with the ultimate goal of TB elimination.

## Introduction

Globally, tuberculosis (TB) is one of the most common infectious diseases and is among the leading causes of death. The World Health Organization (WHO) estimated that there were 10 million new TB cases in the world in 2017 (1). As part of *The End TB Strategy*, the WHO has outlined in *Towards TB Elimination: An Action Framework for Low-Incidence Countries* (i.e., those countries with an incidence rate of 10 TB cases per 100,000 population or fewer), guidance on how to further reduce TB rates to elimination levels (defined as 0.1 cases per 100,000 population) by 2035 (2).

While Canada is a low TB incidence country, TB incidence rates are consistently higher than the low incidence cut-off in certain subpopulations in the country: namely, foreign born and Indigenous Canadians (3). A high level of TB activity in Canada’s north has been observed for many years, especially among the Inuit (3,4). Tuberculosis among foreign born Canadians also represents a large burden of illness in Canada (3). Statistics Canada has projected high growth rates in these two populations in Canada when compared with the population of Canada as a whole (5,6), so it is especially important to diagnose and treat TB, both to address the impact of active TB disease on the affected individual and to prevent any further spread.

In Canada, national surveillance of new and re-treatment cases of active TB is conducted in partnership with all provinces and territories by the Public Health Agency of Canada (PHAC). The primary objective of the Canadian Tuberculosis Reporting System (CTBRS), Canada’s national case based surveillance system, is to monitor and report on the number of cases and on the rates of active TB in Canada. Annual reporting of TB across the country is important to better understand the epidemiology of TB in Canada over time, to inform public health action and to monitor Canada’s progress toward reducing the incidence of TB in Canada, with the ultimate goal of TB elimination (7).

The objective of this report is to provide a descriptive overview of TB cases in Canada in 2017 by age, sex, origin, province/territory and diagnostic classification in the context of previous years’ data. Treatment outcomes for TB cases that were reported to the CTBRS in 2016 are also summarized.

## Methods

The CTBRS maintains non-nominal data on people diagnosed with active TB disease in Canada. Details on the CTBRS's methods, including data collection processes, data management, data quality control and analysis, and the classification and categorization of population subgroups have been described in detail elsewhere (8). In short, provincial and territorial public health authorities voluntarily submit data on all new and re-treatment cases of active TB disease that meet the Canadian case definition for national surveillance (8). Treatment outcome data are submitted between 12 and 18 months following the submission of the initial case report. If treatment is ongoing at the time of data submission to PHAC, the reporting jurisdiction submits an interim report followed by subsequent annual updates until the case file is closed. Updated data from previous years received after the initial submission is reflected in the most current report.

Active TB is classified as either respiratory or non-respiratory. Respiratory TB includes pulmonary TB, TB of the pleura and TB of the intrathoracic or mediastinal lymph nodes, larynx, nasopharynx, nose and sinuses (9). Primary disease is characterized by pleural effusion due to recent (i.e., within the preceding 24 months) infection with *Mycobacterium tuberculosis*. Non-respiratory TB refers to all other disease sites.

Incidence rates in this report were calculated as cases per 100,000 population. Population data used to calculate these rates came from multiple sources. Canadian population data were based on midyear estimates of the Canadian population from Statistics Canada (*unpublished data*). The foreign born population data were based on the 2016 Canadian Census (10). Estimates of the population of Indigenous groups for 2017, namely First Nations, Métis and Inuit, came from the National Household Survey (11). For First Nations persons with status on and off-reserve, population data came from Crown-Indigenous Relations and Northern Affairs Canada’s Indian Registration System as of December 31, 2017 (*unpublished data*).

Original data were maintained according to PHAC’s *Directive for the Collection, Use and Dissemination of Information Relating to Public Health*. Data were cleaned and analyzed using SAS^TM^ Enterprise Guide 5.1 and Microsoft^TM^ Excel 2010. Descriptive findings are presented here. No statistical procedures were used for comparative analyses, nor were any statistical techniques applied to account for missing data. Note that in 2017, British Columbia did not submit information on Indigenous status and, therefore, cases from British Columbia were identified only as either Canadian born or foreign born. Supplementary data tables are available upon request (see **Appendix** for Table list).

## Results

**Figure 1:**
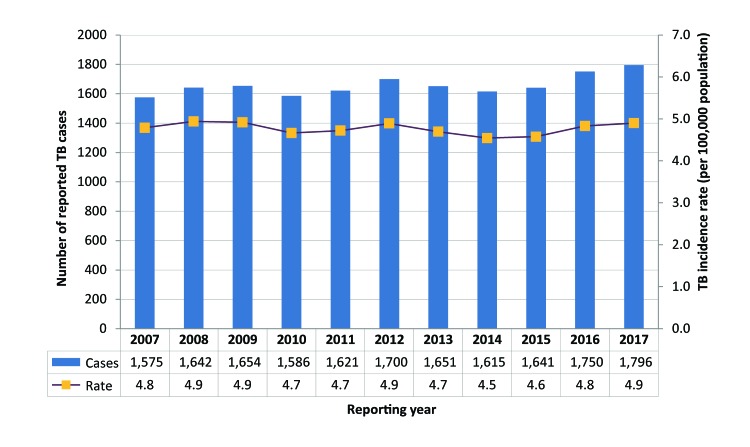
Number of reported tuberculosis cases and incidence rates by year, Canada, 2007–2017 Abbreviation: TB, tuberculosis

There were 1,796 cases of active TB reported in Canada in 2017, compared with 1,750 cases in 2016, representing a 2.6% increase. There was a corresponding increase in the incidence rate from 4.8 to 4.9 per 100,000 population. Of all reported cases in 2017, 92.2% were new cases of active TB and 5.3% were re-treatment cases (i.e., reported having had at least one previous episode of TB). History of previous TB infection was unknown for 2.5% of reported cases. Both the number of cases and the rate of TB in Canada have increased slightly since 2014, when the incidence rate was 4.5 cases per 100,000; however, across the 11 year period, from 2007, the rate increased only slightly (from 4.8 cases per 100,000). The number of TB cases has fluctuated to some extent since 2007, but the absolute number of reported cases in 2017 (1,796 cases) has risen compared with 2007 (1,575 cases) (**Figure 1**).

### Tuberculosis cases by geography

Across Canada, incidence rates of TB varied widely by province/territory in 2017 (**Table 1**). There were no cases of TB reported in Prince Edward Island. Incidence rates of TB were below the national rate of 4.9 per 100,000 population in Newfoundland and Labrador (2.5), Nova Scotia (0.9), New Brunswick (1.1), Quebec (2.6) and Ontario (4.8), but were slightly higher than the national rate in British Columbia (5.3), Alberta (5.3) and the Northwest Territories (6.7). The highest TB incidence rates were in Saskatchewan (8.1), Manitoba (14.0), Yukon (20.8) and Nunavut (265.8). The majority of cases (64.4%) were concentrated in Ontario (37.6%), British Columbia (14.1%) and Alberta (12.6%). While most provinces and territories reported little change from 2016, in Nunavut the number of cases nearly doubled from 2016 to 2017, and the corresponding incidence rate increased from 145.6.0 to 265.8 per 100,000 population (Table 1).

**Table 1:** Number of reported tuberculosis cases and incidence rates per 100,000 population by province and territory, in Canada, 2016–2017

	2016		2017	
Province	Cases	Rate	Cases	Rate
Newfoundland and Labrador	25	4.7	13	2.5
Prince Edward Island	4	2.7	0	0.0
Nova Scotia	3	0.3	9	0.9
New Brunswick	12	1.6	8	1.1
Quebec	252	3.0	217	2.6
Ontario	641	4.6	676	4.8
Manitoba	201	15.2	187	14.0
Saskatchewan	91	7.9	94	8.1
Alberta	238	5.6	227	5.3
British Columbia	225	4.7	253	5.3
Yukon	1	2.7	8	20.8
Northwest Territories	3	6.7	3	6.7
Nunavut	54	145.6	101	265.8
Total for Canada	1,750	4.8	1,796	4.9

### Tuberculosis cases by sex and age

**Figure 2:**
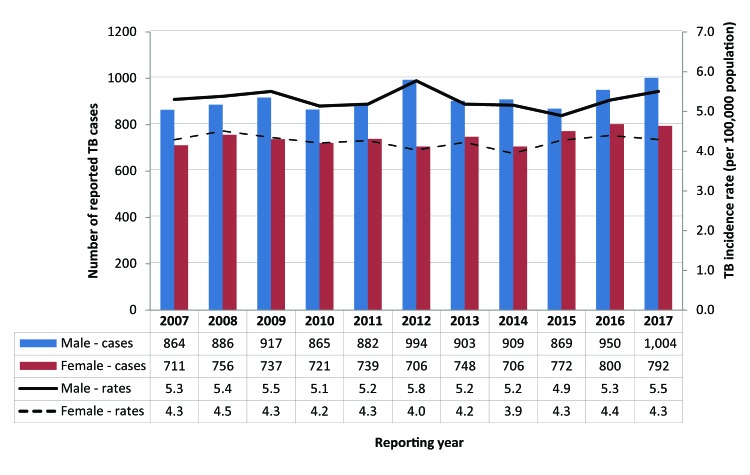
Number of reported cases of tuberculosis and incidence rates by sex and year, Canada, 2007–2017 Abbreviation: TB, tuberculosis

Of the 1,796 reported cases of TB in 2017, 792 (44.1%) were female and 1,004 (55.9%) were male, corresponding to an incidence rate of 4.3 among females and 5.5 among males per 100,000 population. Since 2007, males have consistently accounted for a higher proportion of cases of TB, and correspondingly higher incidence rates (**Figure 2**).

**Figure 3:**
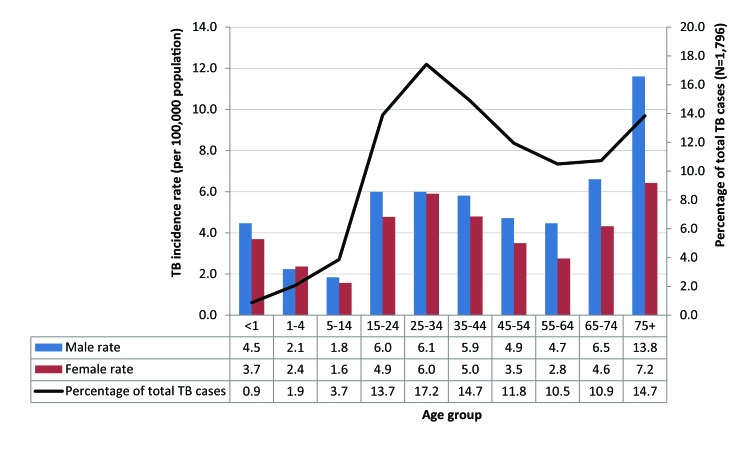
Tuberculosis incidence rates by sex and age group and percentage of tuberculosis cases by age group, Canada, 2017 Abbreviations: N, total number; TB, tuberculosis

Among cases 44 years of age and younger, TB incidence rates among male and female cases were similar; however, after age 44 years, the incidence rate gap between male and female cases begins to widen. In adults aged 75 years and older, the TB incidence rate for male cases was almost twice that of females (13.8 versus 7.2 cases per 100,000) (**Figure 3**).

The highest TB incidence rate was among those aged 75 years and older (10.0 cases per 100,000), followed by those aged 25–34 years (6.0 per 100,000). The lowest incidence rate was among those aged 5–14 years (1.7 per 100,000). The incidence rate among children younger than one year of age increased to 4.1 cases per 100,000 in 2017 from 2.1 per 100,000 in 2016. The largest number of TB cases was in the 25–34 year age group (n=309; 17.2% of total cases) (Figure 3). Children constituted a small proportion of total TB cases in 2017, with infants younger than one year of age accounting for 0.9% of TB cases and children aged one to 14 years accounting for 5.6% of total cases. Trends among TB cases by age have changed little over the past 10 years (3).

### Tuberculosis cases by origin

**Figure 4:**
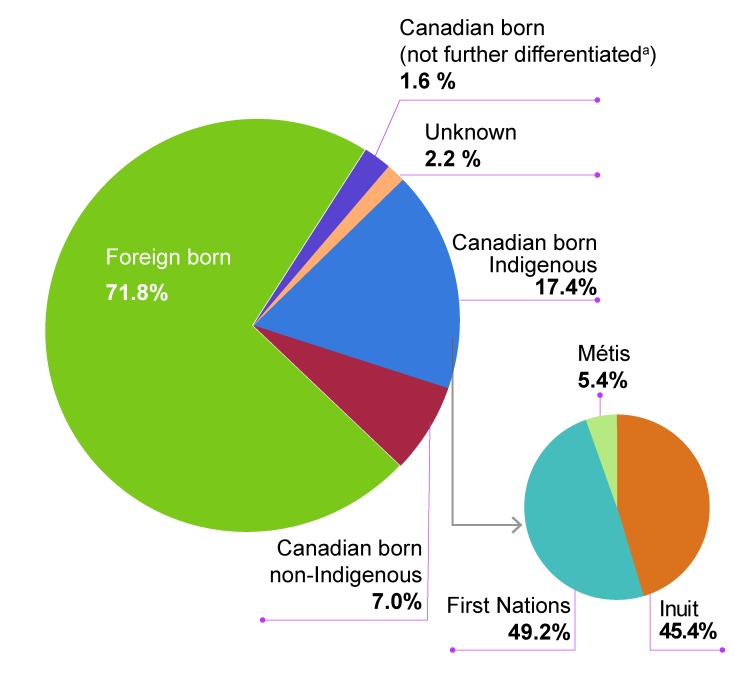
Distribution of tuberculosis cases by origin, Canada, 2017 ^a^ Cases in this group could not be further disaggregated into Indigenous or non-Indigenous

The majority of TB cases reported in Canada in 2017 were foreign born (n=1,290; 71.8%), followed by Canadian born Indigenous cases (n=313; 17.4%) and Canadian born non-Indigenous cases (n=125; 7.0%) (**Figure 4**). An additional 1.6% of cases (n=28) were reported as Canadian born without any further origin breakdown reported, and origin was unknown for a further 2.2% of cases (n=40) (Figure 4).

### Indigenous tuberculosis cases

In Canada in 2017, there were 313 cases of TB reported among Canadian born Indigenous persons, resulting in an incidence rate of 21.5 cases per 100,000 population, a slight decrease from 2016, when the rate was 23.3 cases per 100,000 population (**Table 2**). Of these Indigenous cases, 49.2% (n=154) were First Nations, 45.4% were Inuit (n=142) and 5.4% (n=17) were Métis (Figure 4). Among Métis, the incidence rate of TB was 3.5 cases per 100,000 population, which is lower than the overall Canadian rate (4.9 per 100,000) but higher than the Canadian born non-Indigenous rate (0.5 per 100,000). This rate has generally decreased over time, from 7.5 in 2007 to 2.1 cases per 100,000 in 2016, with a slight rise in 2017 (Table 2). Among First Nations persons, the TB incidence rate in 2017 was 17.1 cases per 100,000 population, which is lower than the rate in 2016 (23.6 per 100,000), however, this rate has fluctuated since 2007 (Table 2). The TB incidence rate in First Nations persons with status living on-reserve in 2017 was 21.7 cases per 100,000, a decrease from the previous year (33.9 per 100,000). Similarly, the incidence rate in First Nations persons with status living off-reserve in 2017 decreased to 9.6 TB cases per 100,000 from 14.5 per 100,000 in 2016 (Table 2). In 2017, there were 142 cases among the Inuit compared to 113 cases in 2016, representing a 25.7% increase. The corresponding incidence rate increased from 168.7 in 2016 to 205.8 cases per 100,000 in 2017. Incidence rates among the Inuit population have ranged from a low of 85.2 per 100,000 population in 2007 to a high of 251.6 cases per 100,000 in 2012, but have consistently been higher than any other population subgroup since 2007 (Table 2).

**Table 2:** Tuberculosis incidence rates and case counts by origin and year, Canada, 2007–2017

Origin		2007	2008	2009	2010	2011	2012	2013	2014	2015	2016	2017
Canadian born non-Indigenous	Cases	171	222	238	185	186	174	159	168	167	140	125
	Rate	0.7	0.9	1	0.7	0.7	0.7	0.6	0.6	0.6	0.6	0.5
Foreign born	Cases	1,067	1,064	1,063	1,054	1,108	1,112	1,153	1,110	1,177	1,224	1,290
	Rate	14.8	14.5	14.4	14.1	14.7	14.6	14.9	14.2	14.9	15.3	14.7
Canadian born Indigenous^a^	Cases	307	347	340	261	303	380	315	320	281	331	313
	Rate	23.9	26.2	24.9	18.6	20.7	24.4	19.9	20	17.1	23.3	21.5
First Nations (FN)^a,b^	Cases	229	232	227	121	177	208	205	182	157	208	154
	Rate	28.4	28.2	26.9	14.1	19.1	21.2	20.3	18	15.2	23.6	17.1
FN living on-reserve	Cases	129	119	122	109	99	113	148	106	101	149	97
	Rate	29.7	26.8	27	23.7	21.2	23.8	30.8	21.7	20.4	33.9	21.7
FN living off-reserve	Cases	83	98	87	73	66	80	50	68	51	56	38
	Rate	24.2	28	24.3	20	16.4	18.7	11.4	15.2	11.1	14.5	9.6
Métis^a^	Cases	32	27	25	26	21	11	18	19	12	10	17
	Rate	7.5	6.1	5.4	5.4	4.4	2.2	3.5	3.6	2.2	2.1	3.5
Inuit^a^	Cases	46	88	88	114	105	161	92	119	112	114	142
	Rate	85.2	160	157.1	200	166.7	251.6	139.4	177.6	164.7	170.1	205.8
Total Canada^c^	Cases	1,575	1,642	1,654	1,586	1,621	1,700	1,651	1,615	1,641	1,750	1,796
	Rate	4.8	4.9	4.9	4.7	4.7	4.9	4.7	4.5	4.6	4.8	4.9

^a^ For 2016 and 2017, BC did not report Indigenous origin of TB cases

^b^ Includes First Nations TB cases where residence on or off reserve was unknown, missing or not reported

^c^ Includes TB cases were origin was unknown, missing or not reported

### Foreign born tuberculosis cases

Similar to previous years, foreign born persons carried the largest burden of TB disease in Canada in 2017 where 71.8% of total cases (n=1,290) were foreign born, corresponding to an incidence rate of 14.7 cases per 100,000 population. Although the incidence rate of TB among foreign born persons in Canada has remained relatively stable since 2007 (n=14.8 per 100,000 population), the absolute number of foreign born cases has steadily increased (Table 2). The number of foreign born cases increased from 1,224 in 2016 to 1,290 in 2017; however, the corresponding incidence rate decreased from 15.3 to 14.7 per 100,000 population.

Country of birth was reported for 97.4% (n=1,256) of these foreign born cases. Similar to 2016, the most commonly reported countries of origin among foreign born TB cases were the Philippines (n=276; 21.4%), India (n=262; 20.3%), China (n=186; 14.4%), Vietnam (n=60; 4.7%) and Pakistan (n=46; 3.6%).

Immigration status at the time of TB diagnosis was known for 63.3% (n=816) of foreign born TB cases reported in 2017. Of these, 77.7% (n=634) were Canadian citizens or permanent residents, 9.6% (n=78) were temporary residents (including students, visitors and workers), and 5.4% (n=44) were refugees, convention refugees and refugee claimants. Immigration status was reported as ‘other’ without further details for 7.4% of cases (n=60).

Year of arrival in Canada was reported for 91.0% (n=1,174) of foreign born TB cases in 2017. Of these, 36.1% (n=424) arrived within the past five years (between 2013 and 2017) and 17.7% (n=208) of these cases were diagnosed with TB within two years of arrival.

### Diagnostic classification

In 2017, diagnostic classification was reported for 1,786 cases (99.4% of total TB cases). Of these, 1,403 (78.6%) were classified as respiratory TB and 383 (21.4%) were non-respiratory TB. Among respiratory TB cases, the most common site of disease was pulmonary (88.3%; n=1,239). Among non-respiratory TB cases, the most common site of disease was the peripheral lymph nodes (50.7%; n=194). By origin, a larger proportion of Canadian born Indigenous cases had respiratory TB (93.9%) compared with Canadian born non-Indigenous (81.5%) and foreign born (74.3%) cases. Similar to previous years, respiratory TB was more common among male cases (83.2%) than female cases (72.7%) and in cases younger than 15 years (91.5%) compared with cases 15 years and older (77.2%).

### Treatment outcomes for 2016

In 2016, 1,750 cases of TB were reported to the CTBRS. Of these, 98.6% (n=1,725) had a TB treatment outcome reported to the CTBRS in 2017. For the majority of these cases (80.2%) treatment was reported as successful (defined as having been cured of TB or having completed TB treatment). Death before or during treatment was reported for 7.6% of cases, where TB was reported to have contributed to or was the cause of death in approximately 60% of these cases. Treatment was reported as ongoing for an additional 5.3% of cases, and 3.0% of cases transferred out of the reporting treatment jurisdiction during treatment. Cases that were lost to follow-up or stopped treatment as a result of an adverse event comprised 0.6% of outcomes and treatment outcome was reported as unknown for 2.3% of cases.

TB treatment success was highest among Inuit and Métis cases, where 92.8% of Inuit cases and 88.9% of Métis cases were reportedly cured or had completed treatment. Treatment success rates were similar among First Nations (78.3%), foreign born (79.1%) and Canadian born non-Indigenous TB cases (78.0%).

## Discussion

In 2017 there was a 2.6% increase in the number of reported cases of TB compared with 2016, and a corresponding increase in the incidence rate from 4.8 to 4.9 per 100,000 population. Since 2014, both the number of cases and incidence rate of TB have steadily increased. Despite these increases in recent years, the incidence rate has changed little since 2007, when it was 4.8 per 100,000 population. In 2017, Nunavut continued to have the highest rate of TB in Canada at 265.8 cases per 100,000, a rate which is nearly 70 times the national rate, whereas the highest concentration of cases was in Ontario (37.6%). While TB rates among foreign born individuals have been fairly stable in the last decade, this population continued to account for the majority of cases in 2017 at 71.8%. Among First Nations persons, the incidence rate of TB in 2017 (17.1 cases per 100,000) declined from 2016 (23.6 per 100,000); however, it was similar to 2015 (15.2 per 100,000) and 2014 (18.0 per 100,000). The rate among Inuit people in Canada in 2017 (205.8 cases per 100,000) was the highest it has been since 2012 (251.6 per 100,000). In 2017, an increase was also seen in the absolute number of cases reported among the Inuit compared to 2016, increasing from 113 to 142 cases in 2017. The majority of TB cases in Canada in 2017 were reported as respiratory TB (78.6%), with pulmonary TB (69.4%) being the most commonly reported diagnostic classification. For the majority of TB cases reported in 2016, TB treatment was successful (80.4%), reflecting effective treatment and high treatment adherence.

Based on these surveillance data alone, it is not known why there has been an increase in the number of TB cases in Canada over the last few years, but several things may be contributing to this. The increase in both the case count and incidence rate between 2016 and 2017 reflects the increases in both the foreign born and Inuit populations during this time period. While the incidence rate of TB in the foreign born population in Canada has been stable in recent years, the case count has steadily increased. This may, in part, be explained by the overall increase in the volume of migrants to Canada in recent years. Foreign born populations in Canada with latent TB infection (not contagious) can become ill with active TB disease years after the migration process or can become infected with TB during travel back to their countries of origin. Stressful living conditions, language and cultural barriers, food and housing insecurity are all factors that can increase the likelihood of reactivating a latent TB infection after migration (7). Increases in TB among the Inuit population are also attributable to an increased risk in this population of progressing from latent TB infection to active TB disease and to ongoing transmission of active TB, related to inequitable access to health care and the social determinants of health (4,7). As well, advances in TB detection, diagnosis and treatment have all recently been reported in Canada’s north, which may be contributing to an increase in detection of TB cases among the Inuit (4).

### Limitations

The limitations of the CTBRS have been described in detail previously (8). The CTBRS is a passive surveillance system and relies on receiving reports of active TB cases in Canada that are diagnosed by health care providers across the country and reported to provincial health authorities, and in turn, reported to PHAC. Completeness of case ascertainment and reporting delays are potential issues with this system; however, the WHO estimates that Canada's surveillance system has a case detection rate of 92% (12).

Finally, it is important to recognize that the data in this report are considered provisional and, as it continues to be updated annually, it are subject to change in future TB surveillance reports. If there are discrepancies between the data summarized in this report and provincial and territorial reports, the most recent provincial and territorial report should be used because updated national data may still be pending.

### Conclusion

Tuberculosis surveillance data from 2017 continue to highlight well known trends in the epidemiology of TB in Canada; namely, that Indigenous and foreign born Canadians continue to be disproportionately represented among cases. Annual reporting of Canadian TB surveillance data is an important tool for informing TB prevention and control efforts and for monitoring progress on initiatives related to reducing the burden of TB in Canada, with the ultimate goal of TB elimination.

## Appendix List of supplementary tables

Supplementary tables available upon request to: phac.tb.surveillance.aspc@canada.ca

**Supplementary Table 1****:** Reported new active and re-treatment tuberculosis cases and incidence rate per 100,000 population (all cases)—Canada and provinces/territories: 2007–2017

**Supplementary Table 1M****:** Reported new active and re-treatment tuberculosis cases and incidence rate per 100,000 population (males) —Canada and provinces/territories: 2007–2017

**Supplementary Table 1F****:** Reported new active and re-treatment tuberculosis cases and incidence rate per 100,000 population (females) —Canada and provinces/territories: 2007–2017

**Supplementary Table 2****:** Reported new active and re-treatment tuberculosis cases and incidence rates per 100,000 population, by age group—Canada: 2007–2017

**Supplementary Table 3****:** Reported new active and re-treatment tuberculosis cases and incidence rate per 100,000 population, by age group—Canada and provinces/territories: 2017

**Supplementary Table 4****:** Reported new active and re-treatment tuberculosis cases and incidence rate per 100,000 population, by origin—Canada and provinces/territories: 2017

**Supplementary Table 5****:** Tuberculosis incidence rates per 100,000 population, by origin—Canada, 2007–2017

**Supplementary Table 6****:** Reported new active and re-treatment tuberculosis cases and incidence rate per 100,000 population, by main diagnostic classification; Canada, 2007–2017

**Supplementary Table 7****:** Reported new active and re-treatment tuberculosis cases and incidence rate per 100,000 population, by main diagnostic classification—Canada and provinces/territories, 2017

**Supplementary Table 8****:** Treatment outcome—Canada and provinces/territories: 2016
